# ^68^Ga-NC-BCH Whole-Body PET Imaging Rapidly Targets Claudin18.2 in Lesions in Gastrointestinal Cancer Patients

**DOI:** 10.2967/jnumed.123.267110

**Published:** 2024-06

**Authors:** Changsong Qi, Rui Guo, Yan Chen, Chenzhen Li, Chang Liu, Miao Zhang, Cheng Zhang, Xiaotian Zhang, Xingguo Hou, Bo Chen, Bing Jia, Zhi Yang, Lin Shen, Hua Zhu

**Affiliations:** 1 Department of Early Drug Development, State Key Laboratory of Holistic Integrative Management of Gastrointestinal Cancers, Beijing Key Laboratory of Carcinogenesis and Translational Research, Peking University Cancer Hospital and Institute, Beijing, China;; 2 Department of Nuclear Medicine, NMPA Key Laboratory for Research and Evaluation of Radiopharmaceuticals (National Medical Products Administration), State Key Laboratory of Holistic Integrative Management of Gastrointestinal Cancers, Beijing Key Laboratory of Carcinogenesis and Translational Research, Peking University Cancer Hospital and Institute, Beijing, China;; 3 Medical Isotopes Research Center, Department of Radiation Medicine, School of Basic Medical Sciences, Peking University, Beijing, China; and; 4 Chengdu AlpVHHs Co. Ltd., Chengdou, China

**Keywords:** ^68^Ga-NC-BCH, PET, CLDN18.2, gastrointestinal cancers

## Abstract

^68^Ga-labeled nanobody (^68^Ga-NC-BCH) is a single-domain antibody–based PET imaging agent. We conducted a first-in-humans study of ^68^Ga-NC-BCH for PET to determine its in vivo biodistribution, metabolism, radiation dosimetry, safety, and potential for quantifying claudin-18 isoform 2 (CLDN18.2) expression in gastrointestinal cancer patients. **Methods:** Initially, we synthesized the probe ^68^Ga-NC-BCH and performed preclinical evaluations on human gastric adenocarcinoma cell lines and xenograft mouse models. Next, we performed a translational study with a pilot cohort of patients with advanced gastrointestinal cancer on a total-body PET/CT scanner. Radiopharmaceutical biodistribution, radiation dosimetry, and the relationship between tumor uptake and CLDN18.2 expression were evaluated. **Results:** ^68^Ga-NC-BCH was stably prepared and demonstrated good radiochemical properties. According to preclinical evaluation,^68^Ga-NC-BCH exhibited rapid blood clearance, high affinity for CLDN18.2, and high specific uptake in CLDN18.2-positive cells and xenograft mouse models. ^68^Ga-NC-BCH displayed high uptake in the stomach and kidney and slight uptake in the pancreas. Compared with ^18^F-FDG, ^68^Ga-NC-BCH showed significant differences in uptake in lesions with different levels of CLDN18.2 expression. **Conclusion:** A clear correlation was detected between PET SUV and CLDN18.2 expression, suggesting that ^68^Ga-NC-BCH PET could be used as a companion diagnostic tool for optimizing treatments that target CLDN18.2 in tumors.

## Visual Abstract

**Figure:**
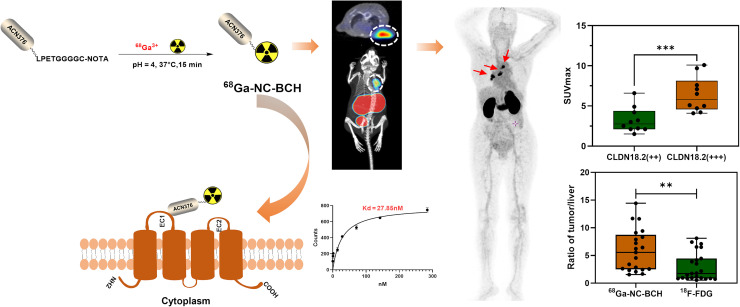

Gastrointestinal cancer is a global disease that seriously endangers human public health. In 2018, gastrointestinal cancers accounted for approximately 4.8 million new cases of cancer and 3.4 million deaths worldwide, or more than a quarter of the total cancer incidence and more than one third of the total cancer-related mortality, respectively (1). Because of the insidiousness of early symptoms, most gastrointestinal cancers are diagnosed at an advanced stage, often leading to a poor prognosis and increased mortality.

Claudin-18 isoform 2 (CLDN18.2) is a subtype of CLDN18, a member of the tight junction protein family (2). CLDN18.2 is involved in the formation of intercellular adhesion structures, cell polarity control, paracellular transport, tissue permeability regulation, and signal transduction (3). Generally, its expression is strictly limited to the normal gastric mucosa; however, CLDN18.2 can also be abnormally activated during the appearance, metastasis, and invasion of gastrointestinal malignancies such as stomach, colon, pancreatic, esophageal, ovarian, and lung tumors (4–6). The randomized phase IIb study showed that zolbetuximab in combination with epirubicin plus oxaliplatin plus capecitabine improved overall survival and progression-free survival in patients with higher CLDN18.2 expression (>70%) relative to those with lower CLDN18.2 expression (40%–69%) (7). Therefore, the detection of CLDN18.2 expression levels is essential for identifying patients who can achieve greater clinical benefit.

To date, there is no standard test for CLDN18.2, and most detection methods involve immunohistochemistry (8). Immunohistochemistry is an invasive process that covers only a small amount of tissue and does not reflect the heterogeneity of CLDN18.2 expression within the tumor. Previously, we reported the first—to our knowledge—clinical results of the ^124^I-labeled CLDN18.2 humanized monoclonal antibody ^124^I-18B10(10L), which showed that CLDN18.2 can be detected in tumor lesions (9). However, the large molecular weight of the monoclonal antibodies results in a long imaging cycle that cannot be completed in a day. Single-domain antibodies are the smallest antibody units with complete functions, stability, and binding antigens; a molecular weight of approximately 15 kDa; and a short circulating half-life in the body with rapid blood clearance, allowing for the matching of radionuclides with short half-lives for same-day PET imaging (10*,* 11).

In this study, we constructed the single-domain antibody molecular probe, ^68^Ga-labeled nanobody (^68^Ga-NC-BCH). With the advantages of whole-body PET, we initiated an open-label, single-center, single-arm, first-in-humans phase 0 trial to study the safety, systemic distribution and dosimetry, and CLDN18.2-targeting ability of ^68^Ga-NC-BCH in patients with gastrointestinal tumors. We focused on assessing whole-body physiologic CLDN18.2 expression and tumor uptake and exploring its main relationship with patient prognosis before and after anti-CLDN18.2 therapy using ^68^Ga-NC-BCH.

## MATERIALS AND METHODS

### Cell and Animal Models

The human gastric adenocarcinoma cell AGS was obtained at Peking University Cancer Hospital and Institute. The AGS^CLDN18.2^ cell line was generated by transfection with the full-length CLDN18.2. All animal experiments were performed in accordance with the guidelines of the Peking University Institutional Animal Care and Use Committee (approval number EAEC 2022-01).

### Small-Animal PET/CT Protocol

Normal Kunming mice and AGS^CLDN18.2^/AGS model nude mice were injected with 7.4 MBq of ^68^Ga-NC-BCH via the tail vein (*n* = 3). Then, 10-min static PET scans were acquired at each time point. Imaging was performed with a small-animal PET/CT scanner (Super Nova PET/CT; PINGSENG).

### Patient Enrollment

The study was approved by the Medical Ethics Committee of the Peking University Cancer Hospital and registered at ClinicalTrials.gov (NCT02760225). All patients signed informed consent forms. All procedures performed in studies involving human participants complied with the ethical standards of institutional or national research councils and the 1964 Declaration of Helsinki and its subsequent amendments or similar ethical standards.

### ^68^Ga-NC-BCH PET/CT Scanning

All imaging was performed on a whole-body PET/CT uEXPLORER scanner (United Imaging). No specific preparation was required for patients. An intravenously administered dose of ^68^Ga-NC-BCH (101.99 ± 44.65 MBq; range, 56.61–177.97 MBq) was used. Five patients underwent dynamic PET/CT imaging. A low-dose CT scan (120 kV, 50 mA, 5-mm slices) was first performed, and then the dynamic PET acquisition was started upon injection of the tracer.

## RESULTS

### Synthesis and Characterization of ^68^Ga-NC-BCH

The molecular weight of the obtained primary single-domain antibody, CLDN18.2-targeting nanobody (ACN376), was defined as 16,075 atomic mass units. The molecular weight of ACN376-GGGGC was determined to be 15,380 atomic mass units (Supplemental Figs. 1A, 1B, and 1D; supplemental materials are available at http://jnm.snmjournals.org). Maleimidomonoamide-NOTA was site-specifically conjugated to ACN376-GGGGC via the maleimide-thiol reaction (Supplemental Fig. 2A). NOTA-ACN376 was obtained with an average NOTA–to–single-domain antibody ratio of approximately 1:1 (Supplemental Fig. 1C). ^68^Ga-NC-BCH was produced with a radiochemical yield of more than 95% and a radiochemical purity of more than 98%. The in vitro stability of ^68^Ga-NC-BCH in 0.01 M phosphate-buffered saline and 5% human serum albumin was demonstrated by a radiochemical purity of more than 98% over 6 h (Supplemental Fig. 3). The quality control results are shown in Supplemental Table 1.

### Saturation Binding Affinity and Cellular Uptake

The binding potency of ^68^Ga-NC-BCH to the CLDN18.2 protein was detected with a dissociation constant of 27.85 nmol/L (Supplemental Fig. 2B). Flow cytometry experiments revealed that 80.2% of the cells in the AGS^CLDN18.2^ group were positively stained with anti-CLDN18.2 antibody (1D5; 1D5 refers to the CLDN18.2 monoclonal antibody used for flow cytometry analysis). Western blotting results confirmed that the expression of CLDN18.2 in AGS^CLDN18.2^ cells significantly differed from that in AGS cells, and the relative expression of CLDN18.2 in the AGS^CLDN18.2^ and AGS cell lines was 0.94 ± 0.16 and 0.28 ± 0.06, respectively (*P* = 0.0027; Supplemental Figs. 4A–4C). The results of the cellular experiment showed that uptake of ^68^Ga-NC-BCH in AGS^CLDN18.2^ cells increased in a time-dependent manner (2.79% ± 0.39% at 10 min, 3.43% ± 0.24% at 30 min, 3.89% ± 0.25% at 60 min, and 4.04% ± 0.18% at 120 min), whereas no significant changes were observed in the AGS group (0.96% ± 0.10% at 10 min, 0.68% ± 0.11% at 30 min, 0.63% ± 0.26% at 60 min, and 0.83% ± 0.07% at 120 min). Moreover, an excess of unlabeled ACN376 and TST001 significantly blocked the uptake of ^68^Ga-NC-BCH (3.89% ± 0.25% vs. 0.77% ± 0.12% vs. 0.93% ± 0.09% at 60 min and 4.04% ± 0.18% vs. 0.69% ± 0.11% vs. 1.07% ± 0.25% at 120 min, respectively) (Supplemental Fig. 2C). Uptake of ^68^Ga-NC-BCH by AGS^CLDN18.2^ cells at 120 min was 4.93-fold greater than that by AGS cells and 5.86-fold greater than that by the blocking group.

### Small-Animal PET/CT Imaging and Immunohistochemistry Study

High uptake in the stomach was observed via static ex vivo imaging and a PET/CT image of Kunming mice spanning 1 h (Supplemental Fig. 5).

**FIGURE 1.**
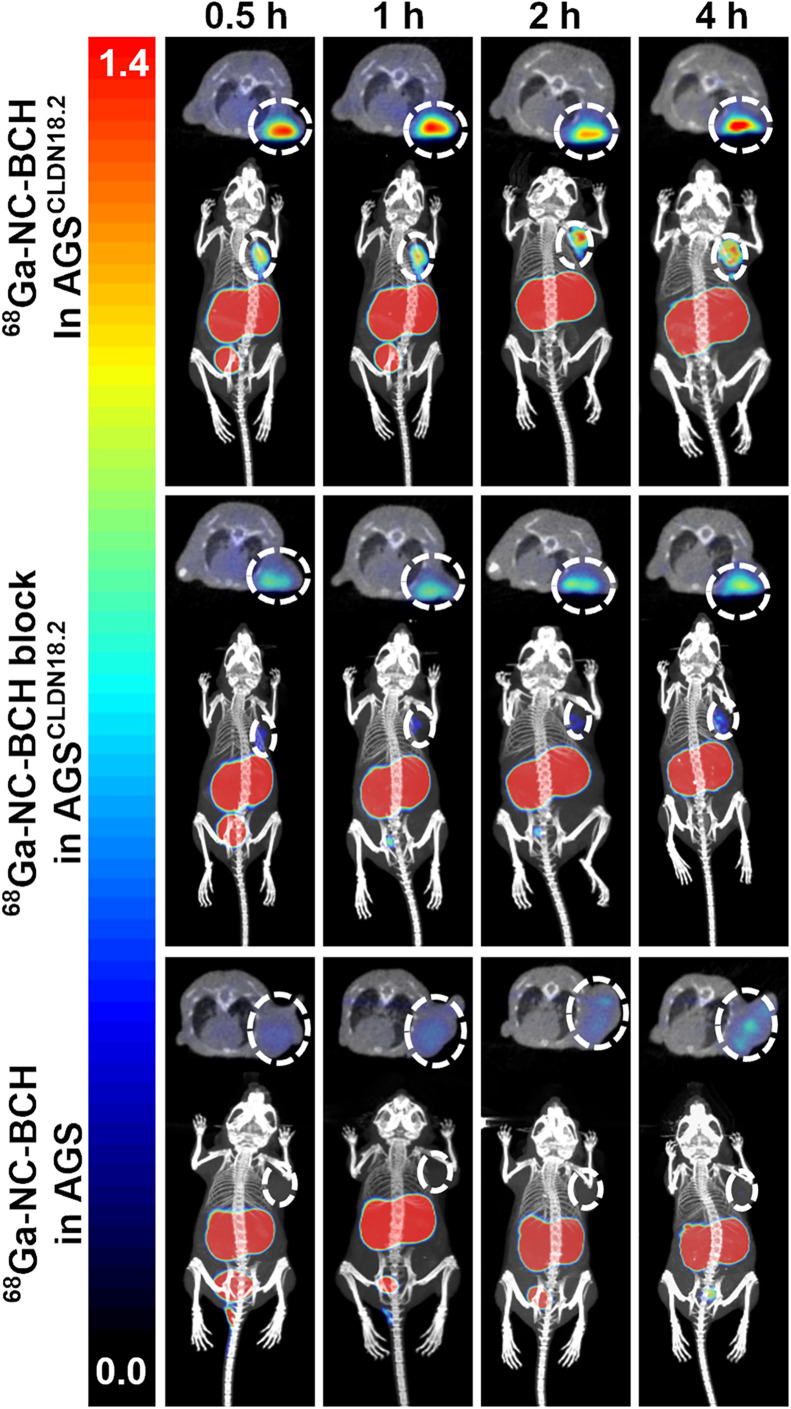
^68^Ga-NC-BCH PET images of mice bearing AGS^CLDN18.2^ and AGS tumors. Block = incubation with 1 mg of TST001 in mice bearing AGS^CLDN18.2^ xenografted 24 h in advance.

Small-animal PET/CT images of AGS^CLDN18.2^ tumor–bearing mice, AGS^CLDN18.2^ tumor–bearing mice pretreated with the antibody TST001 (1 mg) for 24 h, and AGS tumor–bearing mice were obtained at 30, 60, 120, and 240 min after injection of ^68^Ga-NC-BCH (Fig. 1). The kidney SUV_mean_ at 1 h after injection was 14.56 ± 0.29 in the AGS^CLDN18.2^ group, 11.87 ± 0.16 in the AGS^CLDN18.2^ blocking group, and 8.80 ± 0.32 in the AGS group. The SUV_mean_ of the stomach at 1 h after injection was 2.43 ± 0.09 in the AGS^CLDN18.2^ group, 1.65 ± 0.03 in the AGS^CLDN18.2^ blocking group, and 1.67 ± 0.01 in the AGS group. The SUV_mean_ in the AGS^CLDN18.2^, AGS^CLDN18.2^ blocking, and AGS groups at 1 h after injection was 1.14 ± 0.01, 0.54 ± 0.01, and 0.42 ± 0.03, respectively. The tumor-to-muscle ratios at each time point after injection of ^68^Ga-NC-BCH were significantly greater than those of the other control groups, and at 2 h after injection, the tumor-to-muscle ratio reached its maximum of 34.86 ± 4.68 (Supplemental Fig. 6A). The results of immunohistochemistry revealed high and homogeneous CLDN18.2 expression in AGS^CLDN18.2^ tumors, whereas AGS xenograft tumors were negative for CLDN18.2. The gastric mucosa of AGS^CLDN18.2^ and AGS tumor–bearing mice showed substantially positive expression of CLDN18.2 (Supplemental Fig. 6C).

### Biodistribution, Pharmacokinetics, and Safety Study

The biodistribution of ^68^Ga-NC-BCH in AGS^CLDN18.2^ and AGS tumor–bearing mice and the pharmacokinetics study in Kunming mice are presented in Supplemental Figure 6. At 2 h after injection, the stomachs of the mice in all 3 groups exhibited relatively high uptake (6.04 ± 0.66 percentage injected dose [%ID]/g in the AGS^CLDN18.2^ group, 6.36 ± 1.43 %ID/g in the AGS group, and 4.96 ± 0.04 %ID/g in the blocking group). The uptake value of the kidney was higher than that of the stomach (194.86 ± 5.56 %ID/g in the AGS^CLDN18.2^ group, 128.79 ± 0.64 %ID/g in the AGS group, and 129.70 ± 8.55 %ID/g in the blocking group). Tumor uptake in the AGS^CLDN18.2^ tumor–bearing mice was greater (6.61 ± 0.41 %ID/g) than that in the AGS group (0.39 ± 0.13 %ID/g) and the blocking group (1.69 ± 0.84 %ID/g). The pharmacokinetics study showed that ^68^Ga-NC-BCH was cleared quickly from the blood, with a half-life of 22.77 min. After the injection of excess ^68^Ga-NC-BCH (18.5 MBq, 925 MBq/kg), no obvious toxicity was observed in terms of body weight (Supplemental Fig. 7), blood biochemical parameters (Supplemental Table 2; Supplemental Fig. 8), or hematoxylin and eosin staining of main organ tissue slides (Supplemental Fig. 9).

### ^68^Ga-NC-BCH Dosimetry and Biodistribution

Between July 2022 and November 2022, 11 patients were enrolled: 10 with advanced gastric cancer and 1 with advanced colon cancer. Among all patients, the expression of CLDN18.2 was 40% or above. Patient characteristics are shown in Table 1. No tracer-related adverse events were observed in any patients after injection of ^68^Ga-NC-BCH.

**TABLE 1.** Patient Characteristics

Patient no.	Age (y)	Sex	Weight (kg)	Clinical stage	CLDN18.2-targeted therapy before PET
1	60	M	61	cT4aN+M1	No
2	30	F	54	cT3N3M1	No
3	68	F	58	cT4bN2M1	No
4	40	M	69	cT4N2M1	No
5	51	F	52	T3N2M0	No
6	37	M	59	pT2N1Mx	Yes
7	41	M	60	cT4bNxM1	Yes
8	31	F	41	pT4aN3a	No
9	28	F	47	T4aN3aM1	Yes
10	31	F	43	pT3N1M1	Yes
11	37	F	55	cT4aN2M0	Yes

All 11 patients underwent ^68^Ga-NC-BCH PET/CT. The injection dose of ^68^Ga-NC-BCH was 101.99 ± 44.65 MBq (range, 56.61–177.97 MBq). The highest organ dose values for ^68^Ga-NC-BCH were estimated to be for the kidneys, gallbladder, stomach, spleen, and liver. The effective dose of ^68^Ga-NC-BCH was estimated to be 0.042 ± 0.02 mSv/MBq (Supplemental Table 3), which was lower than the radiation dose used for conventional ^18^F-FDG PET/CT (7.0–14.0 mSv) (12). Whole-body PET reduces the total radiation dose and facilitates translational research on this new type of radiopharmaceutical.

**FIGURE 2.**
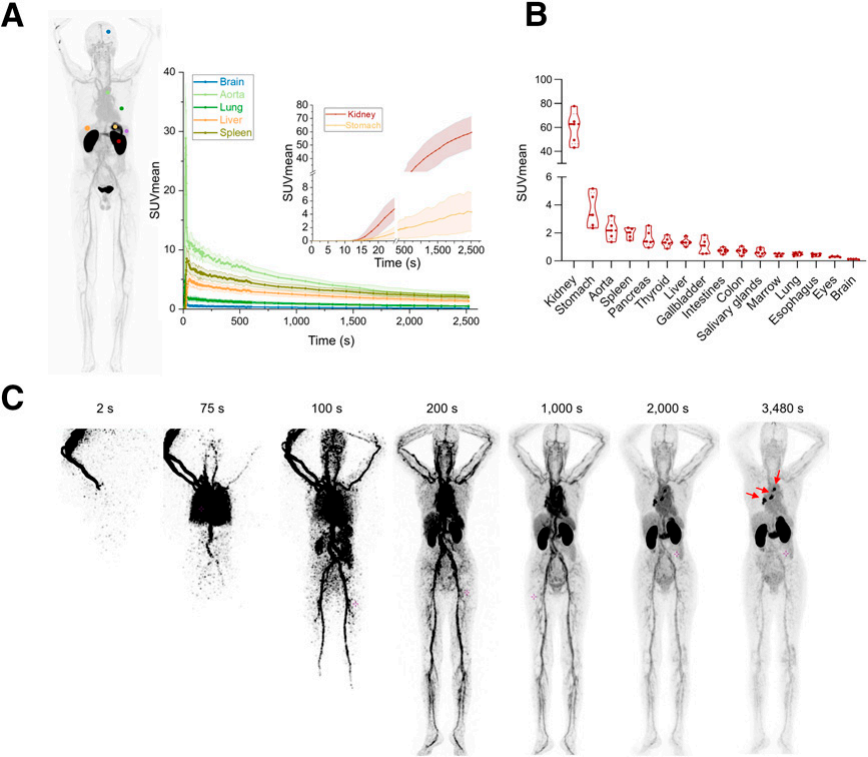
(A) Dynamic changes in SUV_mean_ of selected organs at 0–42 min (*n* = 5). (B) Rank ordering of ^68^Ga-NC-BCH uptake in different organs indicated by SUV_mean_ at 42 min (*n* = 5). (C) Pilot translational study on dynamic total-body PET/CT imaging of ^68^Ga-NC-BCH illustrating time distribution of radiotracers within tumors to optimize imaging window.

Five patients were subjected to dynamic scanning via total-body full-motion PET/CT scans. A 42-min whole-body static PET/CT scan was selected to determine the SUV_mean_ in the major organs of these 5 participants. The dynamic curve showed that the ^68^Ga-NC-BCH activity in the selected organs increased rapidly and gradually decreased to a steady state over time except in the stomach and kidneys (Fig. 2A). A static total-body PET/CT scan at 60 min was selected to determine the SUV_mean_ of the main organs in all 5 participants (Fig. 2B). Except for the stomach wall, the SUV_mean_ for ^68^Ga-NC-BCH in normal tissues (including the spleen, pancreas, liver, brain, and ovaries) was extremely low, as is consistent with previous reports of immunohistochemistry staining of healthy human tissues (6). Since single-domain antibodies are excreted from the body through the kidneys, the probe exhibited strong renal retention in humans. The SUV_mean_ of the positive lesions gradually increased over time from 0 to 58 min (Fig. 2C). The temporal radioactivity curve of the mediastinal metastatic lymph nodes and stomach wall showed a gradual upward trend, and radiotracer accumulation gradually increased over time (Supplemental Fig. 10B).

Surprisingly, all patients with preserved gastric walls had significantly greater uptake of imaging agents in the gastric mucosa (Supplemental Fig. 10A). To our knowledge, this was the first study in which whole-body PET has shown stomach accumulation of a radioactive tracer targeting CLDN18.2 in humans.

### Tumor ^68^Ga-NC-BCH Uptake and Correlation

**FIGURE 3.**
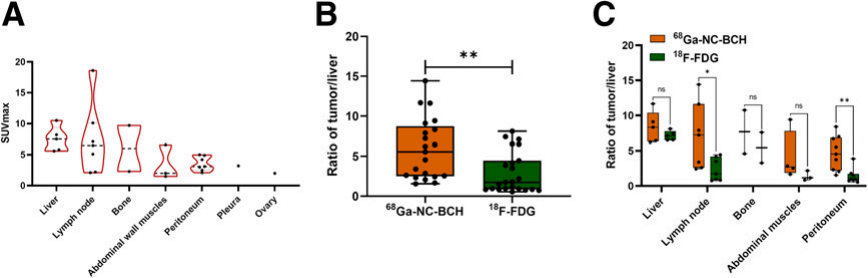
(A) Tumor uptake of ^68^Ga-NC-BCH in different metastatic lesions. (B) Comparison of tumor-to-liver ratio between ^68^Ga-NC-BCH and ^18^F-FDG. (C) Comparison of tumor-to-nontumor ratio between ^68^Ga-NC-BCH and ^18^F-FDG in different metastatic lesions. *P < 0.05. **P < 0.01. ns = not statistically significant.

**FIGURE 4.**
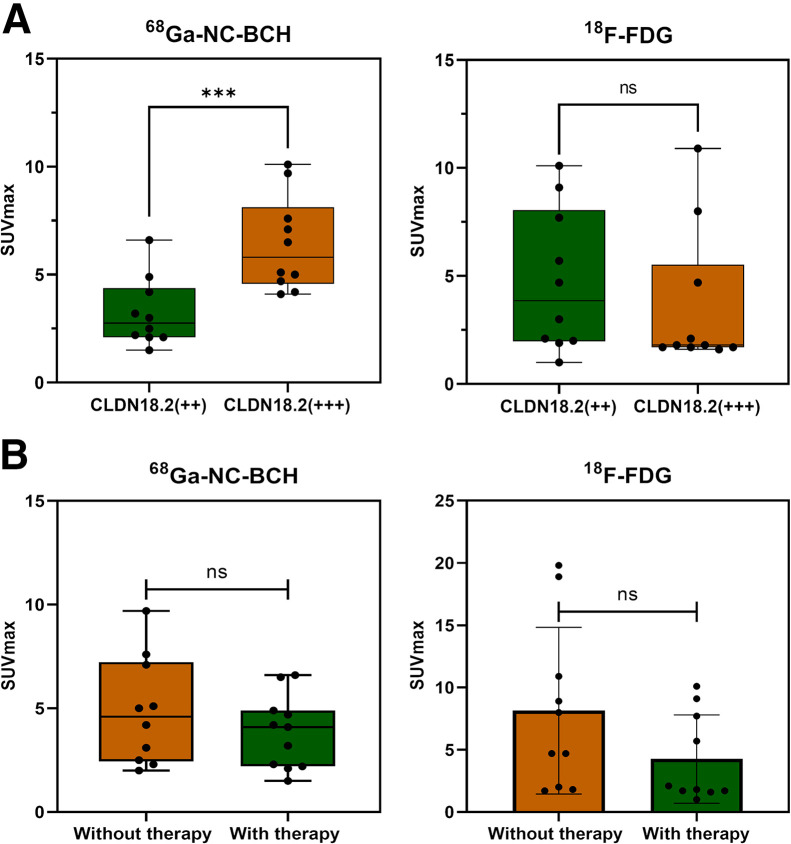
(A) Box plot of ^68^Ga-NC-BCH SUV_max_ and ^18^F-FDG SUV_max_ for all lesions in 11 patients with CLDN18.2 +++ and CLDN18.2 ++ by immunohistochemistry staining. (B) Box plot of ^68^Ga-NC-BCH for all lesions in 11 patients with CLDN18.2-targeted therapy and without CLDN18.2-targeted therapy. Data are mean ± SD.***P < 0.001. ns = not statistically significant.

In total, 215 CLDN18.2-positive lesions were detected in 9 (9/11, 81.8%) patients by ^68^Ga-NC-BCH PET/CT, including 4 patients who had not received CLDN18.2-targeted therapy before imaging and 5 patients who had. The mean SUV_max_ of positive lesions detected by ^68^Ga-NC-BCH PET/CT did not significantly differ among different locations (Fig. 3A). Positive lesions were detected in all patients who had received CLDN18.2-targeted therapy before imaging; these lesions were distributed in the lymph nodes, peritoneum, abdominal muscle, and subcutaneous tissue. The other 4 patients, who had not received CLDN18.2-targeted therapy, had positive lesions distributed among the lymph nodes, peritoneum, liver, bone, and ovaries. The mean SUV_max_ of positive lesions in patients without prior CLDN18.2-targeted therapy was significantly greater than that in treated patients (Fig. 4B).

**FIGURE 5.**
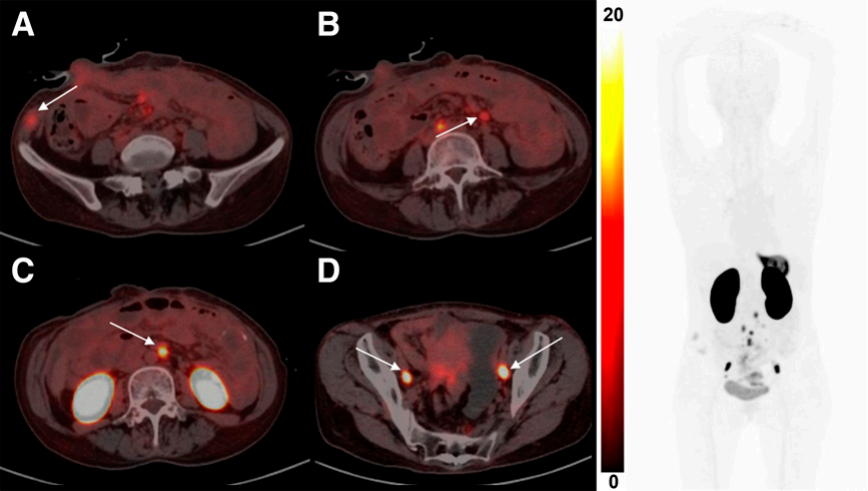
(A) Muscle metastases in right lower abdominal wall with mild elevated uptake. (B–D) Multiple lymph node metastasis showing CLDN18.2 expression degree from low (B) to high (D) (left) and imaging of ^68^Ga-NC-BCH of patient (right).

The CLDN18.2 expression intensity was ++ or +++ in all patients enrolled in the study. ^68^Ga-NC-BCH–positive lesions were detected in 9 patients, including 4 patients with ++ CLDN18.2 expression and 5 patients with +++ expression. The positive lesions were distributed in the liver, lymph nodes, bone, peritoneum, pleura, abdominal muscle, and ovaries (Table 2). ^68^Ga-NC-BCH uptake was highest in liver metastases, followed by lymph nodes and bone. There were statistically significant differences in SUV_max_ between patients with ++ and +++ CLDN18.2 expression in positive lesions (Fig. 4A). Moreover, there was no significant difference in the SUV_max_ of the lesions that were positive on ^18^F-FDG PET/CT regardless of the expression intensity of CLDN18.2 or whether the patients had received CLDN18.2-targeted treatment. Moreover, CLDN18.2 expression differed among metastatic lesions from the same patient (Fig. 5).

**TABLE 2.** Comparison of SUV_max_ of ^68^Ga-NC-BCH with ^18^F-FDG in Metastases

Patient no.	Tumor type	CLDN18.2 expression	Time between prior anti-CLDN18.2 therapy and experiment (mo)	Prior therapy lines	Gastrectomy	Metastatic lesion	^68^Ga-NC-BCH	^18^F-FDG
1	Gastric cancer	90%, 3+	**—**	3	Yes	Peritoneum	5	4.7
						Bone	9.7	19.8
						Liver	7.6	18.9
						Lymph node	7.1	10.9
2	Gastric cancer	80%, 3+	**—**	3	No	Lymph node	Negative	8.9
3	Gastric cancer	40%, 2+	**—**	3	No	Peritoneum	2.5	2
						Lymph node	18.6	4.7
4	Colorectal cancer	40%, 3+	**—**	3	No	Peritoneum	3.1	2.1
						Lymph node	5.1	1.7
5	Gastric cancer	90%, 3+	**—**	3	No	—	Negative	Negative
6	Gastric cancer	50%, 2+	10.5	3	Yes	Anastomosis	4.2	7.7
						Lymph node	2.2	9.1
						Peritoneum	2.1	10.1
						Subcutaneous tissue	1.5	5.7
7	Gastric cancer	90%, 3+	3	3	No	Lymph node	6.5	Negative
						Abdominal wall	2.3	1.7
						Peritoneum	4.1	1.7
8	Gastric cancer	90%, 3+	**—**	3	No	Peritoneum	4.2	Negative
						Bone	2.3	8
						Ovary	2.0	1.8
9	Gastric cancer	40%, 2+	5.5	3	Yes	Peritoneum	4.9	1
						Subcutaneous tissue	6.6	2.1
						Pleura	3.2	Negative
10	Gastric cancer	90%, 3+	15	2	Yes	Peritoneum	4.7	1.8
						Lymph node	10.1	1.6
						Muscle	2.0	Negative
11	Gastric cancer	60%, 2+	6.5	3	No	Peritoneum	3	3
						Lymph node	2.1	1.9

### ^68^Ga-NC-BCH PET/CT and ^18^F-FDG PET/CT

**FIGURE 6.**
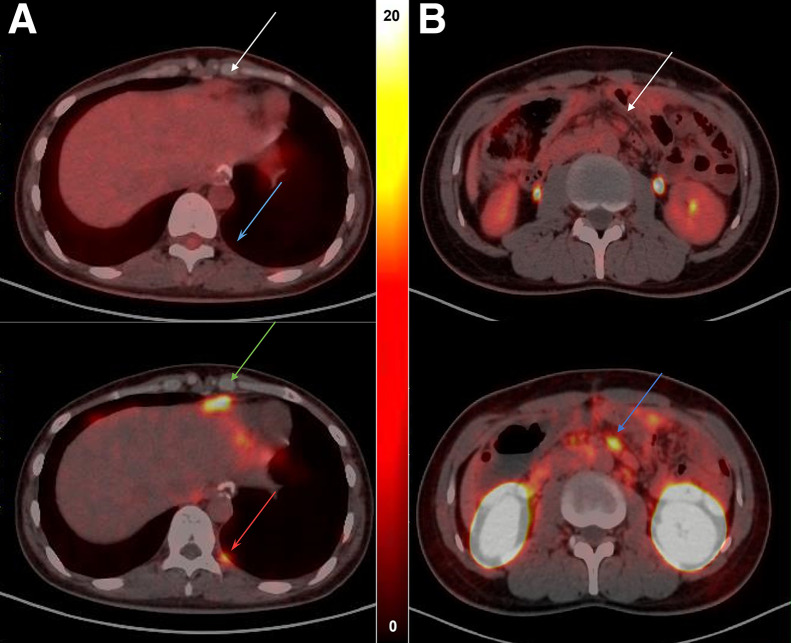
(A) Patient 9 was woman with advanced gastric cancer whose CLDN18.2 expression level was 40%, 2+. Left pleural metastatic nodule (blue arrow) and thickened peritoneum (white arrow) did not show obvious uptake on ^18^F-FDG PET/CT, whereas ^68^Ga-NC-BCH PET/CT showed high uptake (SUV_max_, 3.1 [red arrow] and 5.7 [green arrow]). (B) Patient 4 was man with colon cancer whose CLDN18.2 expression level was 40%, 3+. On ^18^F-FDG PET/CT, abdominal metastatic lymph node (white arrow) showed mild uptake, whereas same lymph nodes on ^68^Ga-NC-BCH PET/CT (blue arrow) showed high uptake (SUV_max_, 1.4 vs. 5.6).

In total, 225 lesions were positive on ^68^Ga-NC-BCH PET/CT, distributed in the liver, lymph nodes, bone, peritoneum, pleura, abdominal muscle, and ovaries. In total, 209 lesions were positive on ^18^F-FDG PET/CT, distributed in the liver, lymph nodes, bone, peritoneum, abdominal muscle, and ovaries. There was a significant difference in the tumor-to-nontumor (T/NT) ratio between all positive lesions detected by the 2 methods. According to a subgroup analysis of the T/NT ratio comparing metastatic lesions at different sites, the T/NT ratio of the lymph nodes and peritoneal metastases detected by ^68^Ga-NC-BCH PET/CT was significantly greater than that detected by ^18^F-FDG PET/CT (Figs. 3B and 3C). ^68^Ga-NC-BCH PET/CT was also able to detect small peritoneal and pleural metastases well. Patient 9, a woman with advanced gastric cancer, had a CLDN18.2 expression level of 40%, 2+. The left pleural metastatic nodule and thickened peritoneum did not show obvious uptake on ^18^F-FDG PET/CT, whereas ^68^Ga-NC-BCH PET/CT showed high uptake (SUV_max_, 3.1 and 5.7). ^68^Ga-NC-BCH PET/CT was also effective at detecting the expression of CLDN18.2 in colon cancer metastases. Patient 4, a man with colon cancer, had a CLDN18.2 expression level of 40%, 3+. On ^18^F-FDG PET/CT, abdominal metastatic lymph nodes showed mild uptake, whereas the same lymph nodes on ^68^Ga-NC-BCH PET/CT showed high uptake (SUV_max_, 1.4 vs. 5.6) (Figs. 6A and 6B). Typical cases are shown in Supplemental Figure 11.

**FIGURE 7.**
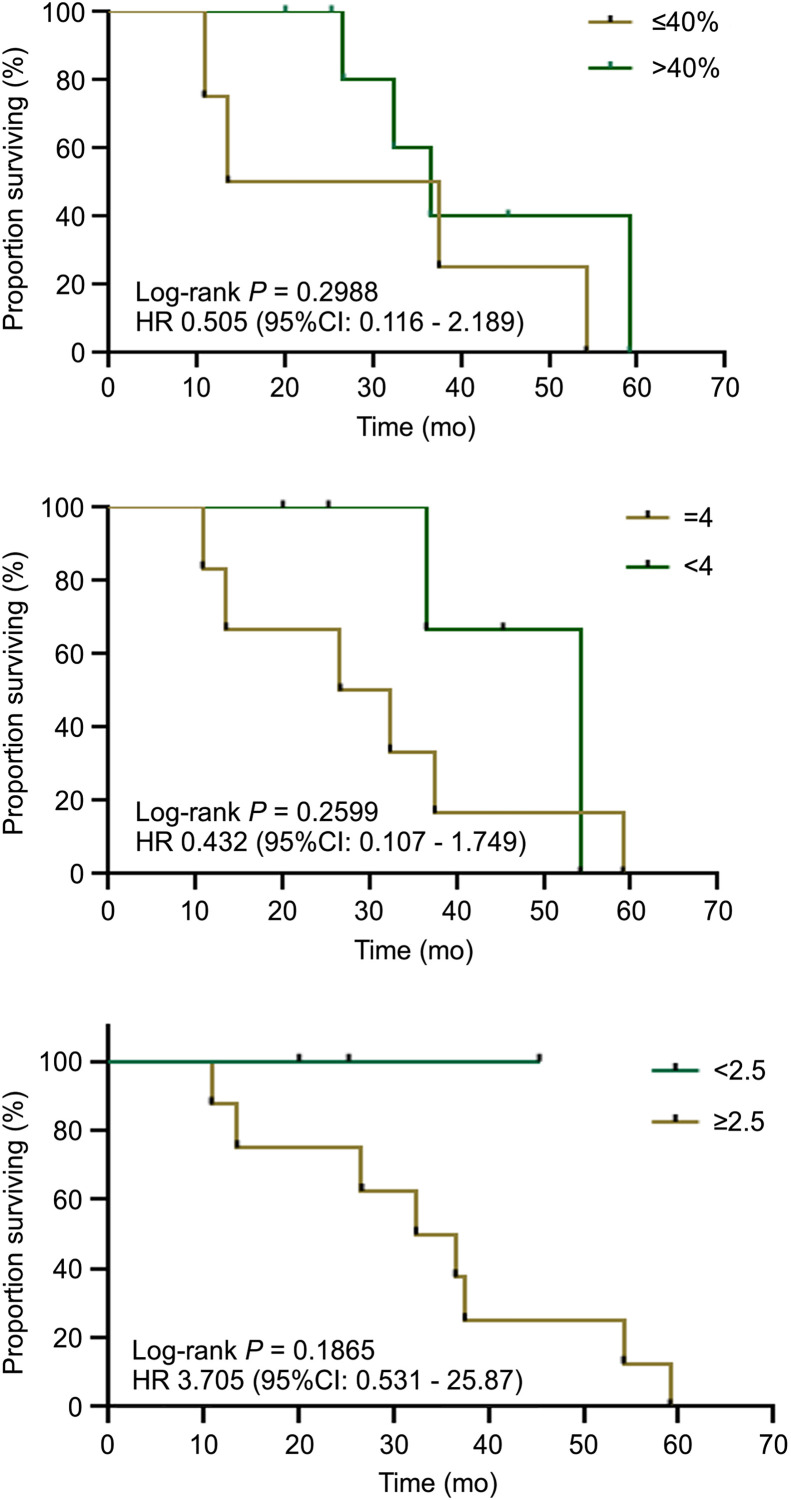
Graphs of survival time. (Top) Green depicts group above—and dark yellow, group below—mean CLDN18.2 expression of 40%. (Middle) Green depicts clinical stage of 4, and dark yellow depicts clinical stage < 4. (Bottom) Green depicts group below—and dark yellow, group above—mean SUV_max_ of 2.5. HR = hazard ratio.

The log-rank method was used to test the difference in survival time distribution between the 2 groups. There was no significant difference in survival time distribution between patients with different CLDN18.2 expression levels (*P* = 0.2988) and no significant difference in clinical stage between the 2 groups (*P* = 0.2599). Moreover, there was no significant difference in survival time between the 2 groups (*P* = 0.1865, SUV_max_ ≥ 2.5 vs. SUV_max_ < 2.5; Fig. 7).

## DISCUSSION

Several forms of CLDN18.2-targeting therapeutic agents, including monoclonal antibodies, antibody–drug conjugates, and chimeric antigen receptor T (CAR-T) cell therapies, are currently undergoing clinical trials worldwide (13*,* 14). Our group previously published interim results of a phase I clinical trial of CLDN18.2-specific CAR-T cells, which showed that the overall response rate and disease control rate of CAR-T cells reached 48.6% and 73.0%, respectively (14). Although CLDN18.2-targeting therapy has achieved good results in clinical studies, we have also noted that the expression level of CLDN18.2 affects therapeutic efficacy to a certain extent.

In this study, we selected a single-domain antibody as a precursor of the radiotracer and labeled it with the short-half-life nuclide ^68^Ga based on its small molecular weight and short cycle time in vivo. Compared with those of traditional antibodies, the rapid tissue penetration and renal clearance rate of single-domain antibodies enable high image contrast to be obtained within 1 h after probe injection, allowing patients to complete the whole imaging workflow within 1 d, greatly increasing compliance and reducing radiation exposure. A preclinical study indicated that ^68^Ga-NC-BCH has good affinity for CLDN18.2 and can specifically bind to CLDN18.2-positive cells. ^68^Ga-NC-BCH was taken up strongly by the gastric mucosa in a mouse model, indicating that the smaller molecular architecture allows it to reach the insidious and dense CLDN18.2 epitope on the gastric mucosa. However, since CLDN18.2 is typically buried in the gastric mucosa, neither of the previous monoclonal antibody-based probes—^89^Zr-DFO-TST001 and ^124^I-18B10(10L)—was available in normal tissue (9*,* 15*,* 16). In addition to gastric organ and positive tumors, probes have a high nonspecific radioactive accumulation in the kidneys, as previously reported (17). The prominent renal uptake is due to excretion of single-domain antibodies through the kidney–urinary system. In addition, nonspecific tubular reuptake after glomerular blood flow is another important factor contributing to elevated renal uptake. Indeed, ^68^Ga-NC-BCH exhibited a longer renal retention time (207.66 ± 19.99 %ID/g, 2 h after injection) than did other single-domain antibody tracers described in previous studies (17–19). The increase in nonspecific radioactive accumulation in the kidneys can lead to high radiation doses in patients and may seriously impede the diagnosis of small perirenal lesions.

Then, we described the results of the first, to our knowledge, ^68^Ga-NC-BCH PET/CT study on patients. According to dosimetry studies, the effective radiation dose of ^68^Ga-NC-BCH was much lower than that of ^124^I-18B10(10L). All results showed that ^68^Ga-NC-BCH PET/CT is a safe, noninvasive imaging method for detecting CLDN18.2 in patients receiving CLDN18.2-targeted therapy.

All 11 patients in the study underwent both ^68^Ga-NC-BCH PET/CT and ^18^F-FDG PET/CT within 1 wk. There was a significant difference in the T/NT ratio between all lesions positive on the 2 methods. Surprisingly, in the subgroup analysis, the T/NT ratio of the lymph nodes and peritoneal metastases detected by ^68^Ga-NC-BCH PET/CT was significantly greater than that detected by ^18^F-FDG PET/CT. Lymph nodes and the peritoneum are the most common metastatic sites of advanced gastric cancer. The T/NT ratio of lesions detected by ^68^Ga-NC-BCH PET/CT was significantly greater, and this high ratio is more conducive to lesion detection. ^68^Ga-NC-BCH PET/CT reflects the expression level of CLDN18.2 in tumor lesions because the tracer is a CLDN18.2-targeting single-domain antibody.

The study included patients who did or did not receive CLDN18.2-targeted therapy before ^68^Ga-NC-BCH PET/CT. Uptake in lesions receiving CLDN18.2-targeted therapy was higher than that in lesions not receiving CLDN18.2-targeted therapy (*P* = 0.2695). This could be because after the first CLDN18.2-targeted therapy, the lesions progressed approximately 1 y later; these advanced lesions still highly expressed CLDN18.2, which also provided a basis for the second CAR-T treatment. More significantly, results showed that metastatic lesions with a higher CLDN18.2 expression level had a higher SUV_max_. This provides some basis for noninvasive detection of CLDN18.2 expression levels in the future.

There were several limitations to the study. First, the small sample size of patients may impede the comprehensive performance of the radiotracer. Also, the small sample challenges us to accurately assess ^68^Ga-NC-BCH uptake in primary gastric tumors because of interference with physiologic uptake in the gastric mucosa and therapeutic response.

## CONCLUSION

We developed a CLDN18.2-specific single-domain antibody nuclide probe that enables same-day PET imaging, and we demonstrated this ability using whole-body PET. Uptake of ^68^Ga-NC-BCH correlated significantly with the expression level of CLDN18.2. ^68^Ga-NC-BCH PET/CT has great potential in the selection of CLDN18.2-targeted therapy strategies and the monitoring of treatment responses by systematically quantifying the systemic expression of CLDN18.2.

## DISCLOSURE

This research was funded by the National Key R&D Program of China (2022YFA0912400), the National Natural Science Foundation of China (82272627, 82171973, and 82171980), Capital’s Funds for Health Improvement and Research (2022-2Z-2154 and 2022-2Z-2155), and Science Foundation of Peking University Cancer Hospital (2022-14). Intellectual properties protection has been filed by Chengdu AlpVHHs Co. Ltd. No other potential conflict of interest relevant to this article was reported.

## Notes

KEY POINTS **QUESTION:** How can CLDN18.2 expression be detected rapidly and accurately in gastrointestinal cancer patients? **PERTINENT FINDINGS:** We demonstrated the feasibility of ^68^Ga-NC-BCH for quantifying CLDN18.2 levels in preclinical models and in patients, in whom a high affinity for CLDN18.2 was detected. **IMPLICATIONS FOR PATIENT CARE:** ^68^Ga-NC-BCH can be used to image CLDN18.2-positive tumors and identify patients with high CLDN18.2 expression as potential candidates for targeted therapy through noninvasive PET imaging.
